# Accelerated dysbiosis of gut microbiota during aggravation of DSS-induced colitis by a butyrate-producing bacterium

**DOI:** 10.1038/srep27572

**Published:** 2016-06-06

**Authors:** Qianpeng Zhang, Yanqiu Wu, Jing Wang, Guojun Wu, Wenmin Long, Zhengsheng Xue, Linghua Wang, Xiaojun Zhang, Xiaoyan Pang, Yufeng Zhao, Liping Zhao, Chenhong Zhang

**Affiliations:** 1 State Key Laboratory of Microbial Metabolism, School of Life Sciences and Biotechnology, Shanghai Jiao Tong University, Shanghai, PR China, 200240; 2 Ministry of Education Key Laboratory for Systems Biomedicine, Shanghai Centre for Systems Biomedicine, Shanghai Jiao Tong University, Shanghai, PR China, 200240.

## Abstract

Butyrate-producing bacteria (BPB) are potential probiotic candidates for inflammatory bowel diseases as they are often depleted in the diseased gut microbiota. However, here we found that augmentation of a human-derived butyrate-producing strain, *Anaerostipes hadrus* BPB5, significantly aggravated colitis in dextran sulphate sodium (DSS)-treated mice while exerted no detrimental effect in healthy mice. We explored how the interaction between BPB5 and gut microbiota may contribute to this differential impact on the hosts. Butyrate production and severity of colitis were assessed in both healthy and DSS-treated mice, and gut microbiota structural changes were analysed using high-throughput sequencing. BPB5-inoculated healthy mice showed no signs of colitis, but increased butyrate content in the gut. In DSS-treated mice, BPB5 augmentation did not increase butyrate content, but induced significantly more severe disease activity index and much higher mortality. BPB5 didn’t induce significant changes of gut microbiota in healthy hosts, but expedited the structural shifts 3 days earlier toward the disease phase in BPB5-augmented than DSS-treated animals. The differential response of gut microbiota in healthy and DSS-treated mice to the same potentially beneficial bacterium with drastically different health consequences suggest that animals with dysbiotic gut microbiota should also be employed for the safety assessment of probiotic candidates.

Butyrate is one of the most important metabolites of the gut microbiota for host health, as it provides the preferential energy source of intestinal epithelium, stimulates the production of regulatory T cells, inhibits inflammation and regulates gene expression as histone deacetylase inhibitor^1^,^2^,^3^,^4^,^5^. All the butyrate we need is produced by butyrate-producing bacteria (BPB) living in our gut^6^. BPB are generally considered to be beneficial members of the gut microbiota, and the depletion of BPB has been associated with inflammatory bowel diseases (IBDs), irritable bowel syndrome (IBS), type 2 diabetes, colorectal cancer, and Parkinson’s disease^7^,^8^,^9^,^10^,^11^. Several BPB have been shown to have anti-inflammatory effects, making them promising candidates for novel probiotics in the treatment of inflammation-related diseases, especially IBDs^12^,^13^.

IBDs are a series of heterogeneous chronic and relapsing inflammatory disorders that affect the digestive tract, and the pathogenesis of IBDs is correlated with dysregulated gut microbiota^14^,^15^,^16^. Compared to those in healthy individuals, the structure and composition of gut microbiota in IBD patients is significantly disrupted, with an increase in certain opportunistic pathogens and a decrease in beneficial bacteria^16^. Emerging studies using culture-independent approaches have demonstrated that one important feature of the gut microbiota from individuals suffering from IBD is the decreased abundance of BPB, such as *Roseburia hominis, Faecalibacterium prausnitzii* and *Eubacterium rectale*^7^,^17^,^18^,^19^. In DSS- or TNBS-induced colitis animal models, BPB strains, such as *F. prausnitzii, Butyricicoccus pullicaecorum, Clostridium butyricum* and *Eubacterium limosum*, have been shown to inhibit myeloperoxidase (MPO) activity and decrease levels of TNF-α and IL-12 in the colon^20^,^21^,^22^,^23^. Thus, the introduction of BPB into the gut microbiota of IBD patients may help alleviate the disease, making them promising candidates for novel probiotics in the treatment of inflammation-related diseases^12^,^13^.

However, as a functional group defined by metabolic potential, BPB exhibit extraordinary diversity and variability^24^,^25^,^26^. BPB are broadly distributed across 10 phyla, and those in the intestine primarily belong to Clostridial clusters IV and XIVa^24^,^26^, which suggests huge variation of metabolic function among BPB strains besides the butyrate production. The impact of novel BPB strains on host health should be rigorously evaluated.

Here we show that one BPB strain isolated from a healthy adult aggravated colitis symptoms and increased the mortality induced by DSS treatment in mice, though it showed potentially beneficial effects to the healthy mice by increasing the butyrate content in the gut without inducing any colitis. We found that the BPB strain accelerated the structural shifts in the gut microbiota induced by DSS treatment, which may have contributed to the aggravation of colitis. Our work shows that potentially beneficial bacterium such as butyrate-producing bacteria may have detrimental effects in diseased animals, showing the need for animal models with dysbiotic gut microbiota to be employed for the safety assessment of these probiotic candidates.

## Results

### *Anaerostipes hadrus* BPB5, a human-derived butyrate-producing bacterium

**Figure 1:**
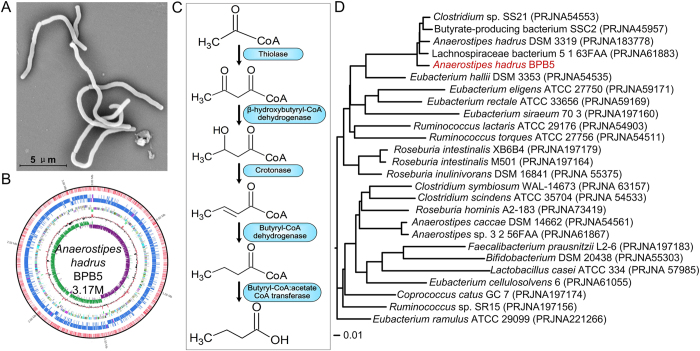
Morphology, phylogenetic location and butyrate-producing pathway of BPB5. (**A**) Electron micrograph of BPB5. (**B**) Complete genome graph of BPB5. From inner to outer: GC skew, GC content, tRNA/rRNA, CDS (reverse and forward strand), m4C and m6A sites in CDS/rRNA/tRNA (reverse and forward). (**C**) Potential butyrate-producing pathway of BPB5. (**D**) CVTree of BPB5 and its neighbours.

BPB5 was isolated from a fresh faecal sample of a healthy human donor by using YCFAGSC medium^27^. Colonies of BPB5 appeared white and opaque with a semi-transparent, irregular margin and were flat and smooth with a diameter of 1–4 mm when grown on YCFAGSC agar after 24 h at 37 °C in our anaerobic workstation (Supplementary Fig. S1A). The BPB5 cells were Gram-positive (Supplementary Fig. S1B), irregularly curved rods (on average, 8 × 0.75 μm in size, Fig. 1A).

When grown in YCFAGSC broth for 24 h at 37 °C in the anaerobic environment, BPB5 produced approximately 10 mM butyrate, which was close to the levels produced by *A. hadrus* DSM 3319^28^. Additionally, the gaseous end products were H_2_ and CO_2_ (Supplementary Table S1). BPB5 produced butyrate when grown with various carbon sources, including monosaccharides, disaccharides and complex prebiotics (Supplementary Table S2). Together, these results indicate that BPB5 is a typical butyrate-producing bacterium.

The nearest neighbour of BPB5 based on 16S rRNA gene was *Anaerostipes hadrus* DSM 3319 (similarity 99.73%) (Supplementary Fig. S1C). These two strains were also clustered on the same branch by the CVTree analysis based on the whole-genome coding sequences (Fig. 1D). The complete genome size of BPB5 was 3.17 M (Fig. 1B) with a G+C content of 37.3 mol%, which was similar to that of *A. hadrus* DSM 3319 (37.2 mol%). C_12:0_ was the dominant component (20.68%) of fatty acids in BPB5 cellular membranes, a value that is also similar to that seen in *A. hadrus* DSM 3319^28^. The genome of *A. hadrus* BPB5 encoded the five essential genes in the bacterial butyrate-producing pathway containing the butyryl-CoA:acetate-CoA transferase gene (*But*) (Fig. 1C), emphasizing its butyrate-producing capacity.

No hits of toxin virulence factors (VFs)-related genes was identified in the genome of *A. hadrus* BPB5. No abnormal response was observed after tail vein injection or oral gavage of BPB5 in specific pathogen free (SPF) mice. Eight hours after the inoculation of 10^9^ BPB5 cells into the mice by gavage, 10^7^ cfu per gram faeces was detected in both healthy mice and DSS-treated mice. BPB5 colonized in the gut of germ-free ICR mice at a level of 10^8^–10^9^ cfu/g faeces, but neither weight loss nor death was observed in the following 7 weeks. These results suggested that BPB5 did not induce acute infection in mice.

### BPB5 increased the butyrate content in healthy mice without great change of gut microbiota structure

**Figure 2:**
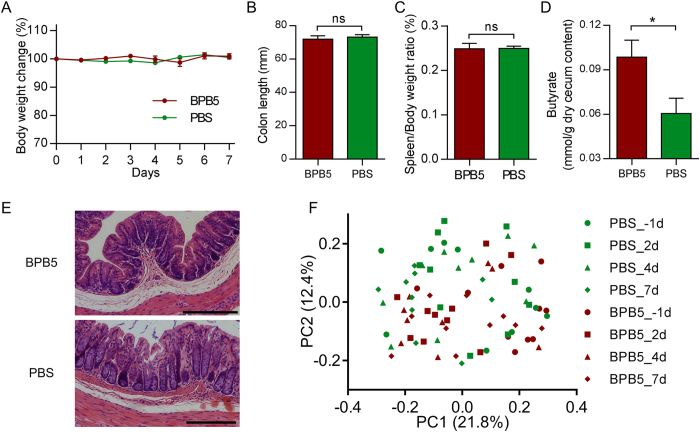
No colitis was induced by BPB5 in healthy mice. (**A**) Body weight change. (**B**) Colon length on Day 7. (**C**) Spleen to body weight ratio on Day 7. (**D**) Butyrate concentration of cecum content on Day 7. (**E**) Hematoxylin-eosin stained sections of distal colon on Day 7. Scale bar: 200 μm. (**F**) Bray-Curtis PCoA plot based on OTU abundance of gut microbiota.

Compare to the PBS group, no significant loss in body weight was observed after the inoculation of BPB5 (Fig. 2A), the colon length, spleen : body weight ratio and histological morphology of distal colon in Hematoxylin-eosin stain did not show any significant difference (Fig. 2B,C,E). After 7 days’ treatment, the butyrate in cecum content was significantly higher in BPB5-inoculated group (Fig. 2D).

To profile the gut microbiota underlying the inoculation of BPB5 in mice, we performed sequencing of the bacterial 16S rRNA gene V3–V4 region on an Illumina MiSeq platform. Principal coordinate analysis (PCoA) based on Bray-Curtis distance showed that the gut microbiota was similar in mice with and without BPB5 gavage (Fig. 2F and Supplementary Fig. S2). And the permutational multivariate analysis of variance (PERMANOVA) confirmed that no significant shift of overall structure on gut microbiota was observed between mice with and without BPB5 gavage (Supplementary Table S3).

### Aggravation of DSS-induced colitis by *A. hadrus* BPB5

**Figure 3:**
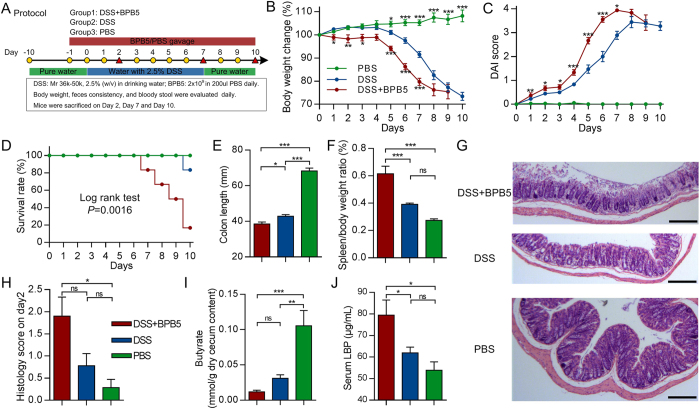
Aggravated DSS-induced colitis in mice inoculated with BPB5. (**A**) Study design. (**B**) Body weight loss. (**C**) DAI score. (**D**) Survival rate. Log rank test was performed for statistics. *P* = 0.0016. (**E**) Colon length on Day 7. For DSS+BPB5 and DSS groups, n = 10; for PBS group, n = 7. (**F**) Spleen weight on Day 7. n = 6 for each group. (**G**) Hematoxylin-eosin stained sections of distal colon on Day 2. Scale bar: 200 μm. (**H**) Histological score on Day 2. For DSS+BPB5 group, n = 10; for DSS group, n = 9; for PBS group, n = 7. (**I**) Butyrate concentration in cecum content on Day 7. For DSS+BPB5 group, n = 5; for DSS and PBS group, n = 7. (**J**) Serum lipopolysaccharide-binding protein (LBP) content on Day 2. For DSS+BPB5 and DSS group, n = 9; for PBS group, n = 6. All data are shown as mean ± s.e.m. One-way ANOVA was used to analyse variation relative to the DSS group at the same time point. *P < 0.05, **P < 0.01, ***P < 0.005, ns, not significant.

Mice treated with DSS exhibited a significant loss in body weight after 5 days relative to PBS controls. Mice in the DSS+BPB5 group showed a significantly greater loss of body weight and higher DAI scores than did animals in the DSS group after Day 1 (Fig. 3B,C). Additionally, the mortality rate was significantly higher in the DSS+BPB5 group than the DSS group. Five out of 12 mice died in the DSS+BPB5 group during the course of treatment, whereas only 1 out of 12 mice died in the DSS group on Day 10 (Fig. 3D). The length of the colon was significantly reduced by DSS treatment, a phenomenon that was further exacerbated by BPB5 inoculation (Fig. 3E). The spleen to body weight ratio in DSS+BPB5 mice was significantly greater than in DSS-treated animals (Fig. 3F). Hematoxylin-eosin staining of distal colon showed broader damage and more severe inflammatory infiltration in the BPB5-inoculated group than in the other two groups after two days’ treatment (Fig. 3G). The histological score was significantly higher in the DSS+BPB5 group than in PBS controls but not the DSS group on Day 2 (Fig. 3H). The butyrate concentration was significantly lower in both DSS+BPB5 and DSS group than PBS group, and it’s much lower in DSS+BPB5 group than DSS group though the difference is not statistically significant (Fig. 3I). Lipopolysaccharide binding protein (LBP) was higher in the DSS+BPB5 group than in the other two groups (Fig. 3J), thus suggesting a higher bacteria-derived antigen burden. The aggravation of DSS-induced colitis by BPB5 was reproduced in two more animal trials (Supplementary Fig. S3).

### Overall structural changes of the gut microbiota in response to DSS and BPB5 treatment

Base on the sequencing data, the richness of the gut microbiota was significantly decreased after 2 days of treatment with DSS (Supplementary Fig. S4A,B). Shannon index revealed a decrease in trend after DSS treatment, which reached significant levels only in BPB5-inoculated mice (Supplementary Fig. S4C,D).

**Figure 4:**
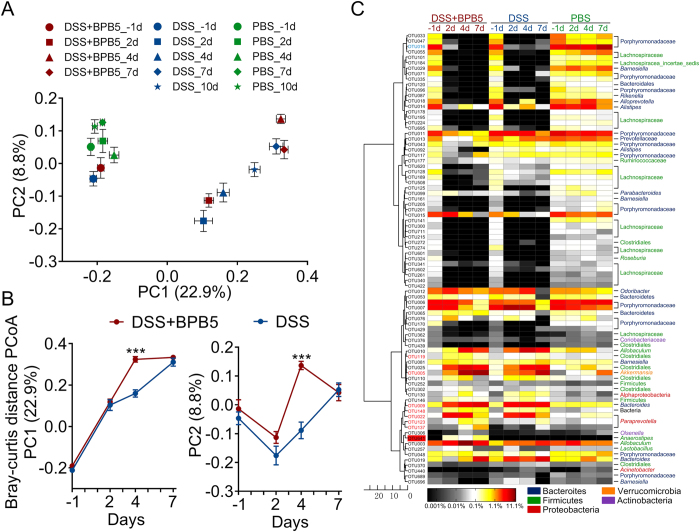
Modulation of gut microbiota by treatment with DSS and BPB5. (**A**) Bray-Curtis PCoA plot based on OTU abundance. (**B**) Variation of gut microbiota structure along PC1 and PC2 of PCoA based on Bray-Curtis distance. Data are plotted as mean ± s.e.m. Mann-Whitney U test was used to analyse variation between two groups. ***P < 0.005. (**C**) Heat map of 83 key OTUs responding to DSS and BPB5 treatment identified by random forest models. The colour of the spots in the panel represents the mean relative abundance (normalized and log-transformed) of the OTU in each group. The values on the colour bar are the relative abundance indicated by the corresponding colour. OTU641 (BPB5) is marked with a red shadow. The OTUs are organized according to their SparCC correlation coefficient. OTUs showing negative correlation (R < −0.5) with OTU366 are marked with blue on the OTU id, whereas positive correlation (R > 0.5) is marked with red. Sample size: for the DSS+BPB5 group, n = 12 on Day -1, n = 11 on Day 2, n = 12 on Day 4, n = 6 on Day 7; for the DSS group, n = 12 on Day -1, 2, 4, n = 6 on Day 7; for the PBS group, n = 12 on Day -1, n = 10 on Day 2, n = 12 on Day 4, n = 6 on Day 7.

PCoA of the Bray-Curtis distance based on OTUs showed the changes of overall structure of gut microbiota in DSS and DSS+BPB5 mice during the DSS challenge (Fig. 4A). The DSS treatment was the predominant factor in shaping gut microbiota, and all samples after DSS treatment were significantly separated from the PBS group (*P* < 0.05 with PERMANOVA test, 9999 permutations, Supplementary Table S4). Although there was no significant difference between these two groups on Day 7, the gut microbiota on Day 2 and 4 were significantly different between the DSS and DSS+BPB5 group, reflecting the impact of BPB5 on the structure of gut microbiota (*P* < 0.01 with PERMANOVA test, 9999 permutations, Supplementary Table S4 and Supplementary Fig. S5). On Day 4, the gut microbiota of the DSS+BPB5 group showed higher variation from baseline than that of mice in the DSS group (Supplementary Fig. S6). According to the scores of PCoA based on Bray-Curtis distance, a greater shift of gut microbiota was observed on Day 4 along both PC1 and PC2 (Fig. 4B). The principal components analysis (PCA) and PCoA based on other distances showed a similar shifting pattern of the gut microbiota (Supplementary Fig. S7). Thus, the overall structure of the gut microbiota was dramatically disrupted by DSS, which was aggravated further and significantly by BPB5.

Random forest models identified 58 OTUs responding to DSS treatment and 29 OTUs responding to BPB5 augmentation (feature accuracy > 0.003, Fig. 4C and Supplementary Fig. S8). In total, 83 non-redundant OTUs contributed significantly to the establishment of the models. At the end of 7 days of treatment, 16 OTUs were significantly enriched by DSS. These OTUs belonged to Bacteroidaceae, Erysipelotrichaceae, Prevotellaceae, *Parasutterella* and *Akkermansia* (Mann-Whitney U test, *P* < 0.05 after Benjamini-Hochberg adjustment, Supplementary Table S5). A total of 55 OTUs were significantly decreased, most of which were species from the families Porphyromonadaceae and Lachnospiraceae (Supplementary Table S5). Among those OTUs, 6 were already significantly changed on Day 2 in the BPB5+DSS group, including 2 OTUs depleted and 4 OTUs enriched (including *Akkermansia* and *Barnesiella*). Of those OTUs, 6 were significantly altered in the DSS+BPB5 group compared to the DSS group on Day 4, including 3 depleted OTUs (Porphyromonadaceae) and 3 enriched OTUs (*Parasutterella* and *Allobaculum*) (Supplementary Table S5).

### Temporal dynamics of gut microbiota members correlated with BPB5

**Figure 5:**
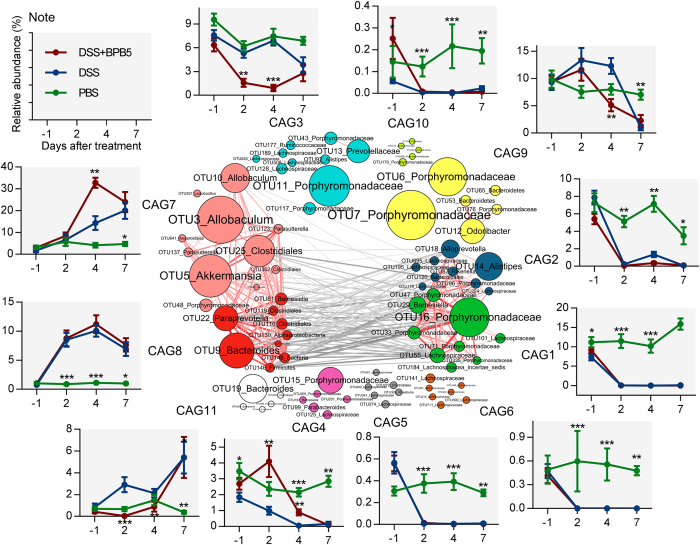
Temporal dynamics in the gut microbiota of mice during the development of DSS-induced colitis. OTU-level network diagram of 83 key OTUs responding to the treatment of DSS and BPB5. Node size indicates the mean abundance of each OTU. Lines between nodes represent correlations between the nodes they connect, with the colour saturation and line width indicating correlation magnitude: red represents positive correlation, grey represents negative correlation. Only lines corresponding to correlations with a magnitude greater than 0.5 are drawn. The OTUs are grouped into 11 CAGs by permutational multivariate analysis of variance (PERMANOVA) when *P* < 0.005. The plots show the abundance of each CAG on Day -1, 2, 4 and 7 in 3 groups. Data in plots represent the total abundance of all OTUs in each CAG from each sample, which were then visualized by mean ± s.e.m. Kruskal-Wallis test was used to analyse variation relative to the DSS group at the same time point. *P < 0.05, **P < 0.01, ***P < 0.005. Sample sizes are the same as Fig. 4C.

To identify the potential interaction among OTUs responding to treatment with DSS and BPB5, the 83 key OTUs were clustered into 11 co-abundance groups (CAGs) based on SparCC correlation coefficients (Fig. 5). CAG7 and CAG8 were positively correlated to form one cluster, and CAG1 and CAG2 formed the other. These two main CAG clusters were negatively correlated with each other.

DSS treatment decreased CAG1, 2, 3, 4, 5, 6, 9 and 10 and enriched CAG7, 8 and 11 (Fig. 5, Supplementary Fig. S9). None of the 11 CAGs were significantly different in abundance between the DSS and DSS+BPB5 groups on Day 7. The abundance of CAG3 and CAG9 was decreased after 7 days of treatment in both the DSS and DSS+BPB5 groups, but a greater and earlier decrease was observed in the DSS+BPB5 group on Day 2 and 4. CAG7 kept increasing throughout the course of treatment, but it showed a greater and earlier surge in the DSS+BPB5 group on Day 4. These results suggest that BPB5 may accelerate the perturbation of gut microbial communities during induction by DSS.

**Figure 6:**
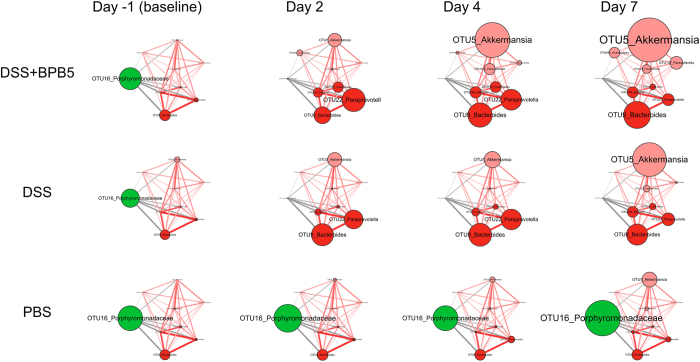
Temporal dynamics of OTUs significantly correlated with BPB5 during the development of DSS-induced colitis. Node size indicates the mean abundance of each OTU. Lines between nodes represent correlations between the nodes they connect, with the colour saturation and line width indicating correlation magnitude: red represents positive correlations, and grey represents negative correlations. Only lines corresponding to correlations with a magnitude greater than 0.5 are drawn. The sample sizes of each group are as described in Fig. 4C.

Among the 83 key OTUs, 8 were significantly correlated with BPB5 in abundance (Figs 4C and 6). One OTU in the families Porphyromonadaceae were negatively correlated with BPB5. A total of 7 OTUs in *Akkermansia, Parasutterella, Bacteroides* and Prevotellaceae showed positive correlation with BPB5.

OTU16, negatively correlated with BPB5, was almost eradicated by the DSS and BPB5 treatments after 2 days. OTUs positively correlated with BPB5 showed different patterns of enrichment after the treatments (Fig. 6). A total of 4 OTUs, including *Akkermansia* (OTU5), *Parasutterella* (OTU123 and OTU137) and OTU119 from Firmicutes were significantly increased in the BPB5-inoculated group at Day 2 or Day 4. However, the enrichment of these OTUs did not occur until Day 7 in the DSS group.

## Discussion

Our current work demonstrated that a human-derived butyrate-producing strain, *Anaerostipes hadrus* BPB5, aggravated DSS-induced colitis in mice, as indicated by more severe and earlier changes in body weight, colon length, spleen weight, histological score, DAI, and mortality rate, though it showed potentially beneficial effects to the healthy mice by increasing butyrate level in the gut. BPB5 is the first BPB to be shown detrimental to host health under a disease-induction condition.

No known virulence factor genes were identified in the genome of BPB5 against the virulence factor database^29^. BPB5 did not induce acute infections in SPF mice after tail vein injection or oral gavage. It colonized the gut of germ-free mice without inducing any colitis or acute infection. Thus, BPB5 did not work as a “pathogen” for aggravating the disease in conjunction with infection by specific pathogens, such as *Helicobacter hepaticus*, which can induce colitis^30^.

Gut microbiota may play an important role in development of colitis, as inflammation induced by DSS is significantly ameliorated in germ-free or antibiotic-treated mice than conventional animals^31^. We then examined the impact of BPB5 on the gut microbiota as a possible mechanism for aggravating the disease.

Alterations to the structure of the gut microbiota are known to occur concurrently with the onset of DSS-induced inflammation^32^,^33^,^34^. In the current work, a dramatic change in overall structure and decrease in diversity of the gut microbiota were observed in DSS-treated mice. DSS-induced structural changes in the gut microbiota are thus a key characteristic closely associated with the development of DSS-induced colitis^34^.

BPB5 alone did not induce significant shift of gut microbiota in the healthy mice, though its augmentation promoted butyrate level in the gut content. However, inoculation with BPB5 shifted the structure of the gut microbiota to the disease stage 3 days earlier than in the group treated with DSS alone, suggesting that the accelerated structural change in the gut microbiota is correlated with a faster disease progression.

After the introduction of BPB5 as an invading species into the gut microbiota, indigenous members, which were correlated with BPB5 in abundance, may be more relevant in the aggravation of the disease^35^. The SparCC algorithm was introduced to identify these phylotypes, which were significantly correlated with BPB5. A total of 4 phylotypes, including *Akkermansia sp.* (OTU5) *and Parasutterella* spp. (OTU123, 137), showed a positive correlation with BPB5 and increased earlier in DSS+BPB5 mice. These bacteria may play an important role in the development of colitis.

Mucin-degrading *Akkermansia* is a commensal bacterium in the mammalian gastrointestinal tract that adheres to the mucus layer and has an important role in maintaining gut barrier function^36^,^37^,^38^. Both Everard and Shin’s work showed that *A. muciniphila* decreased metabolic endotoxemia and diminished the systemic low-grade inflammation induced by high-fat diet without modifying the gut microbiota composition, but the mechanism was not fully uncovered^36^,^39^. However, the enrichment of *Akkermansia* by DSS treatment has been reported in the acute inflammation models of the previous studies using 454 pyrosequencing, quantitative PCR and MITChip^34^,^40^,^41^. Recently, *Akkermansia* has been reported to be increased in faeces of mice with DSS-induced colonic colitis^33^ and patients suffering from colorectal cancer and cholecystitis^42^,^43^. In a gnotobiotic C3H mouse model with a background microbiota of eight bacterial species, infection with *Salmonella enterica* Typhimurium together with *Akkermansia muciniphila* induces more serious inflammation than using *S. enterica* Typhimurium alone^44^. Those suggested that *Akkermansia* may exert different or even opposite physiological function in metabolic syndrome and acute colitis models. In our mouse model, BPB5 induced an earlier explosion of *Akkermansia sp*. (OTU7), which may have contributed to the aggravation of colitis.

Previous studies have shown an increase in pro-inflammatory Gram-negative bacteria during DSS-treatment. The knockout of TLR2 and TLR4 significantly ameliorates DSS-induced colitis, indicating an important role of LPS producers in disease progression^32^,^45^. Gram-negative *Parasutterella* show higher abundance in the gut microbiota of CD patients^46^,^47^. In the current work, *Parasutterella* spp. (OTU123, 137), which were significantly positively correlated with BPB5, increased much earlier than in the DSS-treated group. Serum LBP level was also higher in the DSS+BPB5 group. This finding suggests that the earlier BPB5-modulated increase in LPS producers in the gut microbiota may have also contributed to the aggravation of DSS-induced colitis.

Our findings suggest that a potentially beneficial bacterium for healthy individuals may become an invading species leading to fatal consequences to hosts with disease-disrupted gut microbiota. Similar situation has been recognized about the risk of causing infection or even sepsis in immune-compromised individuals by probiotic *Lactobacillus*^48^. More than 200 cases *Lactobacillus* spp.-associated infections had been reported in patients with ulcerative colitis^49^, short bowel syndrome^50^, cancer *et al*.^51^. Higher mortality was induced by augmentation of a probiotic formula composed of 4 *Lactobacillus* spp. and 2 *Bifidobacterium* spp. in the predicted severe acute pancreatitis patients^52^. Our study not only highlights the important role of the gut microbiota in the progression of inflammatory bowel diseases but also emphasizes the safety precautions that must be taken when BPB are introduced into the guts of IBD patients. Animal models with dysbiotic gut microbiota should be employed for the safety assessment of probiotic candidates.

## Material and Methods

### Isolation and identification of BPB

#### Isolation of BPB5

A fresh faecal sample from a healthy volunteer (male, 44 years old) was transported into an anaerobic work station (DG500, DWS, UK) within 5 min. Written informed consent was obtained from this donor. As in previous studies^27^,^53^, YCFAGSC medium was used to obtain potential BPB isolates.

#### Detection of fermentation products and composition of cellular fatty acids

Substrate utilization was determined as previously described^28^. The SCFA content from the fermentation of BPB5 was quantified as previously described^54^. Gaseous products were measured with a GC7900 gas chromatography work station (Techcomp, China) with a TDX-01 column and TCD detector. The cellular fatty acids of BPB5 were detected with a Sherlock system (MIDI, USA) based on Agilent 7890A with an FID detector and Ultra-2 chromatographic column.

#### Whole-genome sequencing of BPB5

Whole-genome DNA was extracted with a blood and cell culture DNA kit (13343, Qiagen, USA) and sequenced on a PacBio RS II platform. Sequences were assembled into a complete chromosome by the HGAP2.3.0 pipeline^55^. Annotation was performed by GLIMMER Version3.02^56^. We used the CVtree3.0 web server^57^, which applies a composition vector to perform phylogenetic analysis, to construct a phylogenetic tree of BPB5 and reference genomes from the EzTaxon database (http://www.ezbiocloud.net/eztaxon). Blastp was performed based on the whole-genome sequence-translated amino acids of BPB5 against the database of butyrate-producing related genes^26^, requiring a minimum identity of more than 80%. The best hit was used for further analysis. Potential virulence factors (VFs) of BPB5 were identified by blastp against a database of VF-related genes in the toxin super-family of VFDB^29^, with both identity and coverage greater than 50%.

#### Acute pathogenicity test of BPB5 on SPF mice

Acute pathogenicity of BPB5 was tested by injecting 100 ul PBS solution containing 10^8^ cells of BPB5 to specific pathogen free (SPF) C57BL/6 mice (9 week old, n = 4) via tail vein, or inoculating 10^9^ cells of BPB5 to C57BL/6 mice (9 week old, n = 10) through gavage. The response of mice was observed carefully within 2 hours after the injection or gavage.

#### Potential pathogenicity test of BPB5 on Germ-free mice

10^8^ cells of BPB5 in PBS solution was inoculated to Germ-free ICR mice (male, 10–11 week old, n = 7), the colonization state and response of mice was evaluated in the following 7 weeks. The colonization level of BPB5 in the gut of Germ-free mice was semi-quantitatively measured by counting cells under the microscope according to the calibration curve built from pure culture cells of BPB5.

### Evaluating the physiological function of BPB5 in healthy mice and DSS-induced colitis mice

#### Healthy mice

Specific pathogen-free, 7-week-old male C57BL/6 mice were purchased from SLAC Inc. China (Shanghai, China). BPB5 was grown anaerobically in YCFAGSC medium overnight. Then, the cultures were washed and concentrated in phosphate-buffered saline (PBS) with 0.05% (w/v) L-cysteine for a final concentration of 10^10^ colony-forming units (cfu)/mL under strictly anaerobic conditions. This BPB5 solution was kept on ice and used within 1 hour. Bacterial suspensions in anaerobic PBS were administered to mice in the BPB5 group by oral gavage at a dose of 2 × 10^9^ cfu/0.2 mL. Mice in the PBS group were inoculated daily with 0.2 mL PBS. The mice were weighed and visually inspected for diarrhoea and rectal bleeding during the experimental period. Then, the disease activity index (DAI), incorporating weight loss, diarrhoea and bleeding, was calculated based on data collected by double-blinded observers according to standardized scoring^58^.

Fresh faeces were collected on Day -1 (baseline, before BPB5 inoculation), 2, 4 and 7. All faecal samples were stored at −80 °C until analysis. 10 mice from each group were sacrificed on Day 7 and blood, colon contents, colon tissues and caecal content were collected. Mice were humanely euthanized prior to serum and tissue sample collection.

#### DSS-induced colitis mice

Dextran sodium sulphate (DSS; molecular weight, 36,000–50,000, MP) was dissolved in drinking water at a final concentration of 2.5% (w/v) and given *ad libitum* to the mice from Day 0 to Day 7 as shown in Fig. 3A. Mice in the DSS+BPB5 group were inoculated with 2 × 10^9^ cfu/0.2 mL BPB5, other two groups were inoculated daily with 0.2 mL PBS. The preparation of BPB5 and gavage operation were the same as in healthy mice experiment. Fresh faeces were collected on Day -1 (baseline, before BPB5 inoculation and DSS treatment), 2 and 4. For DSS+BPB5 group, DSS group and PBS group, we sacrificed 10, 9 and 7 mice on Day 2 respectively; sacrificed 16, 16 and 13 mice on Day 7; sacrificed 3, 12 and 13 mice at the end of experiment. Blood, colon contents, colon tissues and caecal content were collected right after the sacrificing. Serum lipopolysaccharide binding protein (LBP) was determined after a dilution of 1:1600 using the Mouse Lipopolysaccharide Binding Protein ELISA Kit (Cell Sciences, Canton, MA, USA) according to the manufacturer’s instructions.

The bacterial isolation experiments based on the human faecal sample were approved by the School of Shanghai Jiao Tong University Ethics Committee Biomedical Project (No. 2014-018), and all experiments were performed in accordance with the relevant guidelines and regulations. All operations and experimental methods of the animal experiments were approved by the Animal Care and Use Committee of the School of Life Sciences and Biotechnology, Shanghai Jiao Tong University (No. 2014-005), and carried out according to its guidelines. Three independent sets of experiments using DSS-induced colitis mice model were performed to confirm the results.

#### Detection of BPB5 survived through the digestive tract of SPF mice

Fresh fecal samples were collected 8 hours after inoculation on Day 5. Samples were stored in PBS containing 0.05% L-Cysteine (w/v) on ice until transporting to the anaerobic work station within 1 hour. The BPB5 strain used here was labeled with rifampicin resistance. Plate counting was perform to detect the living cells of BPB5 on YCFAGSC agar with 100 μg/mL rifampicin.

#### Histological analysis

For histological analysis, tissue samples were fixed in 4% paraformaldehyde and then embedded in paraffin. Subsequently, 4 μm sections were stained by hematoxylin-eosin (HE). Histological scoring was performed as previously described^59^.

#### V3–V4 regions in 16S rRNA gene sequencing

DNA extraction from faecal samples was conducted as previously described^60^ and purified with the Omega Gel Extraction kit (D2501-01, OMEGA Bio-Tek, Taiwan, China). A sequencing library of V3–V4 regions of the 16S rRNA gene was prepared as described in http://res.illumina.com/documents/products/appnotes/16s-metagenomic- library-prep-guide.pdf, with some modifications. Platinum Pfx DNA polymerase (C11708021, Invitrogen, USA) was used for two steps of amplification. For the Amplicon PCR (amplification of 16S rRNA V3–V4 region), the 25-ul reaction mix consisted of 1.6x Pfx amplification buffer, 1 mM MgSO4, 0.3 mM dNTP, 0.2 μM of each specific primer for V3 to V4 region of 16S rRNA gene as described in protocol, 0.75 U of Platinum Pfx DNA polymerase and 10 ng template DNA. PCR cycles was reduced to 21 to diminish the PCR bias. The program was started with pre-denaturation at 94 °C for 3 min, followed by denaturation at 94 °C for 30 s, annealing at 55 °C for 30 s and extension at 72 °C for 30 s for 21 cycles with a final extension step at 72 °C for 8 min. For the Index PCR (attachment of dual indices and Illumina sequencing adapter using the Nextera XT Index Kit), the 25-ul reaction mix consisted of 1.0× Pfx amplification buffer, 1 mM MgSO4, 0.2 mM dNTP, 2.5 ul of each N7 and S5 Index primers as described in protocol, 0.5 U of Platinum Pfx DNA polymerase, and 2.5 ul purification product of the Amplicon PCR step as template DNA. The PCR program of Index PCR was the same as Amplicon PCR except reducing the cycle number to 8. The purification and Index PCR were both carried according to the protocol. The purified products were mixed at equal ratio for sequencing using the Illumina MiSeq System (Illumina Inc., USA).

#### Analysis of sequencing data

Both the forward and reverse ends of the same read were truncated at the first base where the Q value was no more than 2. If the pair of reads had a minimum overlap of 50 bp, they were then merged into a complete read. These reads were not kept unless longer than 399 bp with an expected error of no more than 0.5^61^. Two batches of sequencing data from healthy and DSS-induced colitis mice were pooled before OTU picking. Quality-filtered reads were dereplicated into unique sequences and then sorted by decreasing abundance, and singletons were discarded. Representative non-chimeric OTU sequences were next picked by Uparse’s default^62^. Further reference-based chimera detection was performed using UCHIME^63^ against the RDP classifier training database (v9)^64^. The OTU table was finalized by mapping quality-filtered reads to the remaining OTUs with the Usearch^61^ global alignment algorithm at a 97% cutoff.

The number of high-quality reads of 2 sample were less than 9000, which was removed from further analysis. Then, the sequences of all the samples were downsized to 9000 (1000 permutations) to equal the difference in sequencing depth. All subsequent analysis was performed based on the QIIME platform (version 1.8)^65^. The alpha diversity of each sample was calculated with observed OTUs and the Shannon index. Representative sequences for each OTU were built into a phylogenetic tree by FastTree and subjected to the RDP classifier to determine the phylogeny with a bootstrap cutoff of 80% (RDP database version 2.10). The preliminary results of sequencing on 16S rRNA gene V3–V4 region were presented in the Supplementary Results.

Random forest models^66^ were introduced to identify specific bacterial phylotypes that contributed to the segregation of gut microbiota induced by DSS and/or BPB5. Group pairs with a significant difference (*P* < 0.05, PERMANOVA based on Bray-Curtis distance) were included for random forest discrimination. Models with class error = 0 were considered successful. The importance of an OTU was determined based on the mean decrease in accuracy of discrimination, and OTUs with a value greater than 0.003 were considered key OTUs.

The correlation among 83 key OTUs was calculated by the SparCC algorithm^67^ with a bootstrap procedure repeated 100 times and then visualized into a network diagram. The Ward clustering algorithm and PERMANOVA (9999 permutations, *P* < 0.005) based on SparCC correlation coefficients were used to cluster the 83 key OTUs into 11 co-abundance groups (CAGs) using the R program.

#### Statistical analysis of physiological and microbiota data

To test the statistical significance of the physiological and biochemical data, normally distributed data were assessed by analysis of variance followed by a Tukey *post hoc* test (Prism, GraphPad, California, USA). Differences were considered significant when *P* < 0.05. The Mann–Whitney U test and Kruskal-Wallis test were used to analyse the variance of microbiota data, and *P* values were adjusted as described by Benjamini and Hochberg^68^. Differences were considered significant when FDR < 0.05.

#### Sequence data accession numbers

The 16S rRNA gene sequence information in this study has been submitted to the GenBank Sequence Read Archive database under accession number SRP060584. The whole-genome sequence of *Anaerostipes hadrus* BPB5 has been submitted to NCBI under accession number CP012098.

## Additional Information

**How to cite this article**: Zhang, Q. *et al*. Accelerated dysbiosis of gut microbiota during aggravation of DSS-induced colitis by a butyrate-producing bacterium. *Sci. Rep.* **6**, 27572; doi: 10.1038/srep27572 (2016).

## Supplementary Material

Supplementary Information
