# Supplementary material for: Accelerated dysbiosis of gut microbiota during aggravation of DSS-induced colitis by a butyrate-producing bacterium
Source: Sci Rep. 2016 Jun 6;6:27572. doi: 10.1038/srep27572 (PMC4893749; doi:10.1038/srep27572)
Supplement: Supplementary Information [file srep27572-s1.pdf]

Accelerated dysbiosis of gut microbiota during aggravation of DSS-induced colitis by a butyrate-producing bacterium

Qianpeng Zhang, Yanqiu Wu, Jing Wang, Guojun Wu, Wenmin Long, Zhengsheng Xue, Linghua Wang, Xiaojun Zhang, Xiaoyan Pang, Yufeng Zhao, Liping Zhao and Chenhong Zhang

Supplementary Information

Files in this Data Supplement

Supplementary results
Supplementary Table S1
Supplementary Table S2
Supplementary Table S3
Supplementary Table S4
Supplementary Table S5
Supplementary Figure S1
Supplementary Figure S2
Supplementary Figure S3
Supplementary Figure S4
Supplementary Figure S5
Supplementary Figure S6
Supplementary Figure S7
Supplementary Figure S8

Supplementary Figure S9

Supplementary Results

Summary of sequencing on 16S rRNA gene V3-V4 region

On average, 16415 ±3148 (mean ±s.d.) high-quality reads were obtained for each sample. A total of 716 operational taxonomic units (OTUs) were delineated at a threshold of 97% identity using the UPARSE pipeline. Only one OTU (OTU641) was identified as Anaerostipes sp., which was detected only in the samples from mice inoculated with BPB5. Of the total reads, 99.4% were assigned to 9 different phyla, including Firmicutes (45.1%), Bacteroidetes (41.6%), Proteobacteria (7.8%) and Verrucomicrobia (3.1%).

Supplementary Figures and Tables

Supplementary Table S1 Changes in fermentation products (mM) of BPB5 after growth for 24 h in YCFAGSC medium

Strain	BPB5
Acetate	-2.73
Propionate	-0.52
Isobutyrate	-0.08
Butyrate	10.81
Isovalerate	-0.08
Valerate	-0.04
CO_2	0.50
H_2	0.70

Supplementary Table S2 Carbon source spectrum of BPB5

Growth activity	Substrates
Positive growth (OD>0.4)	glucose, sucrose, raffinose, trehalose, sorbierite, D-galactose, D-fructose, fructo-oligosaccharide, galacto-oligosaccharides, arabinose, mannitol, L-sorbose, inulin.
Weak growth (0.4>OD>0.15)	starch, xylose, glycerol.
No growth (OD<0.15)	cellobiose, maltose, melibiose, L-rhamnose, xylan, dextrin, ribose, esculin, nutriose

Supplementary Table S3 Impact of BPB5 on microbiota in healthy mice

	P **- value[a]**
BPB5_2d *vs*. PBS_2d	0.04978
BPB5_4d *vs*. PBS_4d	0.30800
BPB5_7d *vs*. PBS_7d	0.05091

a: PERMANOVA test based on Bray-Curtis distance of samples followed by FDR adjustment.

Supplementary Table S4 Impact of DSS and BPB5 on segregation of microbiota in DSS-induced colitis mice

	P **- value[a]**	**Class error[b]**
DSS_2d *vs*. PBS_2d	0.00147	0
DSS_4d *vs*. PBS_4d	0.00147	0
DSS_7d *vs*. PBS_7d	0.00396	0
DSS+BPB5_2d *vs*. DSS_2d	0.00147	0
DSS+BPB5_4d *vs*. DSS_4d	0.00147	0
DSS+BPB5_7d *vs*. DSS_7d	0.13960	ND

a: PERMANOVA test based on Bray-Curtis distance of samples followed by FDR adjustment.

b: Leave one out cross validation class error rate of Random Forest models of each group pair. ND no data.

Supplementary Table S5 Taxonomy, dynamics and correlation information of 83 key OTUs.

OTU_ID	CAG group	Taxonomical assignments (RDP Classifier)			*P* - value[a]	R[b]	Day 2[c]	Day 4[d]
		Phylum	Family	Genus				
OTU33	1	Bacteroidetes	Porphyromonadaceae		0.00036 * ↓	-0.45		
OTU47	1	Bacteroidetes	Porphyromonadaceae		0.00036 * ↓	-0.46		
OTU16	1	Bacteroidetes	Porphyromonadaceae		0.00036 * ↓	-0.51 #		
OTU55	1	Firmicutes	Lachnospiraceae		0.00036 * ↓	-0.48		
OTU101	1	Firmicutes	Lachnospiraceae		0.00149 * ↓	-0.30		
OTU184	1	Firmicutes	Lachnospiraceae	Lachnospiracea_incertae_sedis	0.00036 * ↓	-0.28		
OTU29	1	Bacteroidetes	Porphyromonadaceae	*Barnesiella*	0.00748 * ↓	-0.41		
OTU71	1	Bacteroidetes	Porphyromonadaceae		0.01141 * ↓	-0.42		
OTU335	1	Bacteroidetes	Porphyromonadaceae		0.00066 * ↓	-0.37		
OTU120	2	Bacteroidetes			0.00036 * ↓	-0.36		
OTU96	2	Bacteroidetes	Porphyromonadaceae		0.00036 * ↓	-0.39		
OTU87	2	Bacteroidetes	Rikenellaceae	*Rikenella*	0.00036 * ↓	-0.36		
OTU18	2	Bacteroidetes	Prevotellaceae	*Alloprevotella*	0.00036 * ↓	-0.46		
OTU14	2	Bacteroidetes	Rikenellaceae	*Alistipes*	0.00036 * ↓	-0.40		
OTU178	2	Firmicutes	Lachnospiraceae		0.00036 * ↓	-0.27		
OTU195	2	Firmicutes	Lachnospiraceae		0.00036 * ↓	-0.24		
OTU224	2	Firmicutes	Lachnospiraceae		0.00036 * ↓	-0.36		
OTU695	2	Firmicutes	Lachnospiraceae		0.00149 * ↓	-0.28		
OTU11	3	Bacteroidetes	Porphyromonadaceae		0.00506 * ↓	-0.23		↓
OTU13	3	Bacteroidetes	Prevotellaceae		1	-0.29		
OTU43	3	Bacteroidetes	Porphyromonadaceae		0.0172 * ↓	-0.20	↓	↓
OTU92	3	Bacteroidetes	Rikenellaceae	*Alistipes*	0.23559	-0.37		
OTU117	3	Bacteroidetes	Porphyromonadaceae		0.71649	-0.15		
OTU177	3	Firmicutes	Ruminococcaceae		0.00506 * ↓	-0.09		
OTU620	3	Firmicutes	Lachnospiraceae		0.00729 * ↓	-0.21	↓	

OTU_ID	CAG group	Taxonomical assignments (RDP Classifier)			*P* - value[a]	R[b]	Day 2[c]	Day 4[d]
		Phylum	Family	Genus				
OTU128	3	Firmicutes	Lachnospiraceae		0.00238 * ↓	-0.41		
OTU189	3	Firmicutes	Lachnospiraceae		0.00115 * ↓	-0.43		
OTU508	3	Firmicutes	Lachnospiraceae		0.01004 * ↓	-0.35		
OTU125	4	Firmicutes	Lachnospiraceae		0.05659	-0.14		
OTU99	4	Bacteroidetes	Porphyromonadaceae	*Parabacteroides*	0.00115 * ↓	0.03		
OTU15	4	Bacteroidetes	Porphyromonadaceae		0.00187 * ↓	-0.07		
OTU161	4	Bacteroidetes	Porphyromonadaceae	*Barnesiella*	0.01212 * ↓	-0.22		
OTU201	4	Bacteroidetes	Porphyromonadaceae		0.00066 * ↓	-0.22		
OTU205	4	Bacteroidetes	Porphyromonadaceae		0.00491 * ↓	-0.22		
OTU272	5	Firmicutes			0.00149 * ↓	-0.25		
OTU274	5	Firmicutes	Lachnospiraceae		0.00036 * ↓	-0.22		
OTU601	5	Firmicutes	Lachnospiraceae		0.00149 * ↓	-0.21		
OTU602	5	Firmicutes	Lachnospiraceae		0.00149 * ↓	-0.25		
OTU324	5	Firmicutes	Lachnospiraceae	*Roseburia*	0.00491 * ↓	-0.17		
OTU341	5	Firmicutes	Lachnospiraceae		0.00036 * ↓	-0.20		
OTU141	6	Firmicutes	Lachnospiraceae		0.00149 * ↓	-0.16		
OTU340	6	Firmicutes	Lachnospiraceae		0.00491 * ↓	-0.11		
OTU215	6	Firmicutes	Lachnospiraceae		0.00491 * ↓	-0.14		
OTU300	6	Firmicutes	Lachnospiraceae		0.00149 * ↓	-0.09		
OTU711	6	Firmicutes	Lachnospiraceae		0.01212 * ↓	-0.16		
OTU261	6	Firmicutes	Lachnospiraceae		0.02683 * ↓	-0.06		
OTU422	6	Firmicutes	Lachnospiraceae		0.00149 * ↓	-0.01		
OTU10	7	Firmicutes	Erysipelotrichaceae	*Allobaculum*	0.00036 * ↑	0.37		
OTU123	7	Proteobacteria	Sutterellaceae	*Parasutterella*	0.00149 * ↑	0.54 #		↑
OTU25	7	Firmicutes			0.00036 * ↑	0.49	↑	
OTU5	7	Verrucomicrobia	Verrucomicrobiaceae	*Akkermansia*	0.00036 * ↑	0.6 #	↑	

OTU_ID	CAG group	Taxonomical assignments (RDP Classifier)			*P* - value[a]	R[b]	Day 2[c]	Day 4[d]
		Phylum	Family	Genus				
OTU137	7	Proteobacteria	Sutterellaceae	*Parasutterella*	0.00253 * ↑	0.6 #		↑
OTU641	7	Firmicutes	Lachnospiraceae	*Anaerostipes*	1	1 #		
OTU306	7	Actinobacteria	Coriobacteriaceae	*Olsenella*	0.52839	0.48		
OTU302	7	Firmicutes			0.98738	0.49		
OTU257	7	Firmicutes	Lactobacillaceae	*Lactobacillus*	0.65071	0.34		
OTU48	7	Bacteroidetes	Porphyromonadaceae		0.00036 * ↓	0.37		
OTU3	7	Firmicutes	Erysipelotrichaceae	*Allobaculum*	0.01277 * ↑	0.45		↑
OTU110	8	Firmicutes			0.98738	0.40		
OTU252	8	Firmicutes			0.04332 * ↑	0.43		
OTU130	8	Proteobacteria			0.00036 * ↑	0.46		
OTU9	8	Bacteroidetes	Bacteroidaceae	*Bacteroides*	0.00036 * ↑	0.56 #		
OTU146	8	Firmicutes			0.01720 * ↑	0.48		
OTU148	8	un_Bacteria			0.00361 * ↑	0.60 #		
OTU22	8	Bacteroidetes	Prevotellaceae	*Paraprevotella*	0.00115 * ↑	0.53 #		
OTU119	8	Firmicutes			0.01004 * ↑	0.55 #	↑	
OTU81	8	Bacteroidetes	Porphyromonadaceae	*Barnesiella*	0.00036 * ↑	0.47	↑	
OTU12	9	Bacteroidetes	Porphyromonadaceae	*Odoribacter*	0.00036 * ↓	0.03		
OTU76	9	Bacteroidetes	Porphyromonadaceae		0.00036 * ↓	0.13		
OTU65	9	Bacteroidetes			0.00115 * ↓	0.34		
OTU6	9	Bacteroidetes	Porphyromonadaceae		0.00149 * ↓	0.03		
OTU53	9	Bacteroidetes			0.00036 * ↓	-0.18		
OTU7	9	Bacteroidetes	Porphyromonadaceae		0.00238 * ↓	-0.23		↓
OTU170	10	Bacteroidetes	Porphyromonadaceae		0.02683 * ↓	-0.06		
OTU429	10	Bacteroidetes	Porphyromonadaceae		0.43275	0.02		
OTU362	10	Firmicutes	Lachnospiraceae		0.01212 * ↓	0.11		
OTU439	10	Firmicutes			0.02683 * ↓	0.10		

**OTU_ID**	**CAG group**	**Taxonomical assignments (RDP Classifier)**			***P* - value[a]**	**R[b]**	**Day 2[c]**	**Day 4[d]**
		Phylum	Family	Genus				
OTU376	10	Actinobacteria	Coriobacteriaceae		0.08012	0.17		
OTU19	11	Bacteroidetes	Bacteroidaceae	*Bacteroides*	0.00149 * ↑	0.18		
OTU370	11	Firmicutes			0.05659	0.15		
OTU440	11	Proteobacteria	Moraxellaceae	*Acinetobacter*	0.02290 * ↑	0.22		
OTU689	11	Bacteroidetes	Porphyromonadaceae		0.01212 * ↓	0.11		
OTU696	11	Bacteroidetes	Porphyromonadaceae	*Barnesiella*	0.02683 * ↓	0.07		

a: Indicate the difference between abundance of DSS_7d and DSS_-1d, calculated by Mann Whitney test followed by Benjamini and Hochberg adjustment. * $P < 0.05$, ↑ significantly increased in DSS_7d, ↓ significantly decreased in DSS_7d.

b: SparCC correlation coefficient between 83 OTUs and OTU366 (BPB5), # $R > 0.5$ or $R < -0.5$.

c: OTUs that were significantly changed in both DSS_7d and DSS+BPB5_2d, but not in DSS_2d. ↑ significantly increased, ↓ significantly decreased.

d: OTUs that were significantly changed in both DSS_7d and DSS+BPB5_4d, but not in DSS_4d. ↑ significantly increased, ↓ significantly decreased.

Supplementary Figure S1

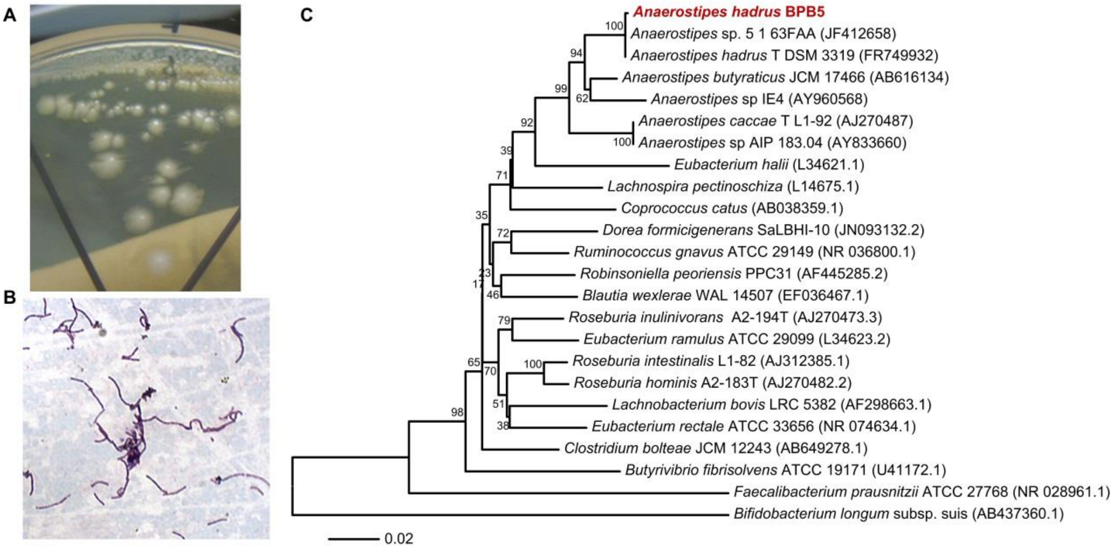

Supplementary Figure S1 Morphology and phylogeny of BPB5. (A) Colonies of BPB5 on YCFAGSC agar after 24 hours growth. (B) Gram stain of BPB5. (C) Phylogenetic tree based on 16S rRNA gene sequence of BPB5 and neighbors. The tree was generated by the neighbor-joining method, with *Bifidobacterium longum* as outgroup. Bar, 2% sequence divergence.

Supplementary Figure S2

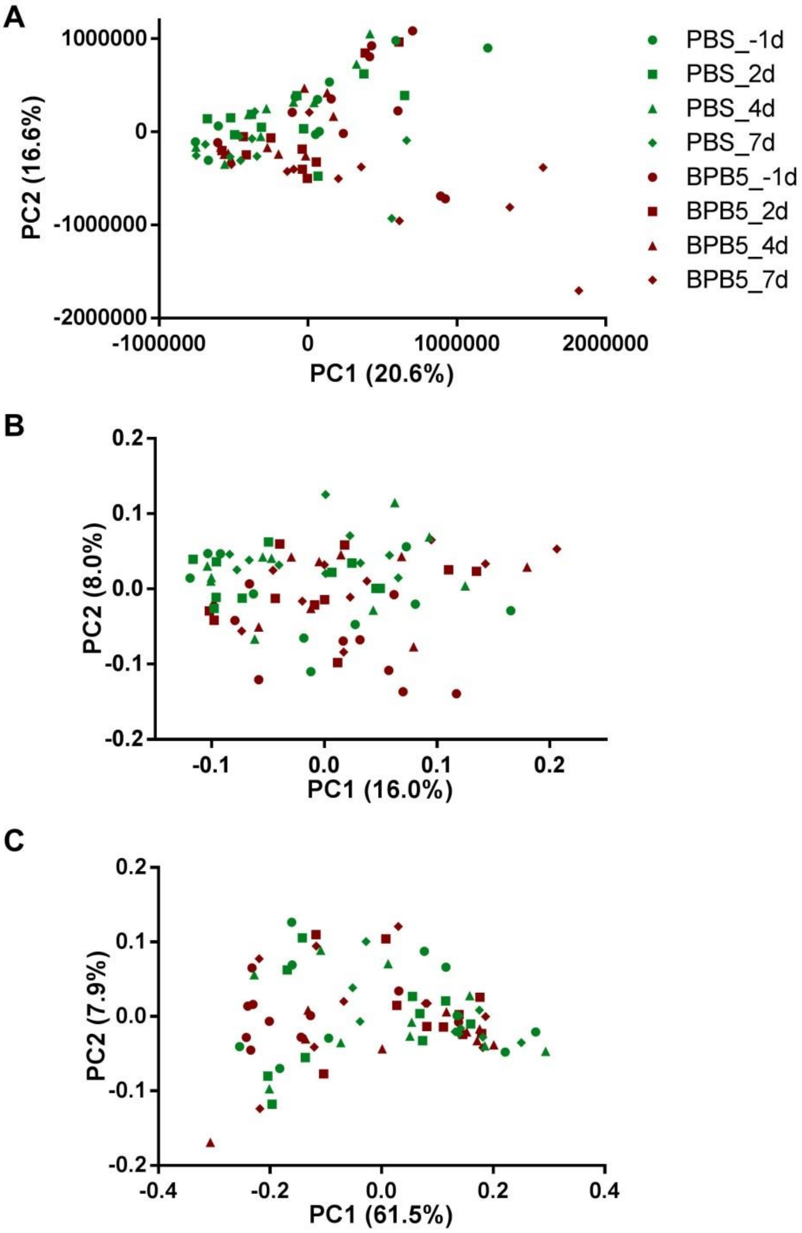

Supplementary Figure S2 Limited structural variation of gut microbiota of the 79 samples included in the study. (A) PCA. (B) Unweighted Unifrac PCoA. (C) Weighted Unifrac PCoA. Sample number of each group is the same as Fig. 2.

Supplementary Figure S3

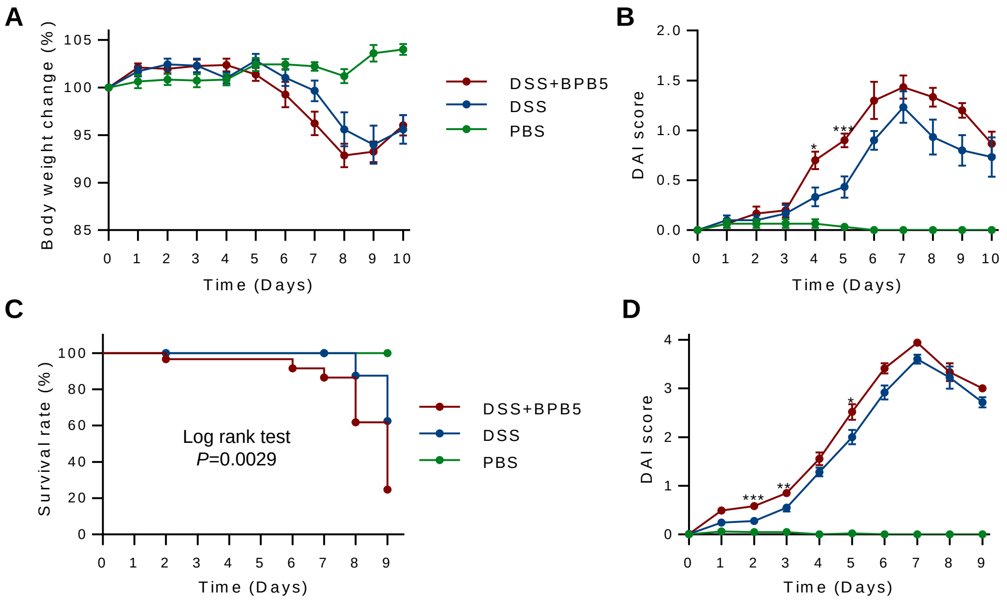

Supplementary Figure S3 The aggravation of DSS-induced colitis by BPB5 In the other two batches of animal trials. (A) and (B) are from batch BUM4, (C) and (D) are from batch BUM8. In BUM4, a moderate colitis was induced by the DSS and BPB5 treatments, and no mice died in the processing. In BUM8, a severe colitis was induced after 7 days' treatment, significantly higher mortality was induced in DSS+BPB5 group than DSS group, *P*=0.0029, Logrank test. All data are shown as mean ± s.e.m. One-way ANOVA was used to analyze variation compared to DSS group at the same time point. * p<0.05, ** p<0.01, *** p<0.005, ns, not significant.

Supplementary Figure S4

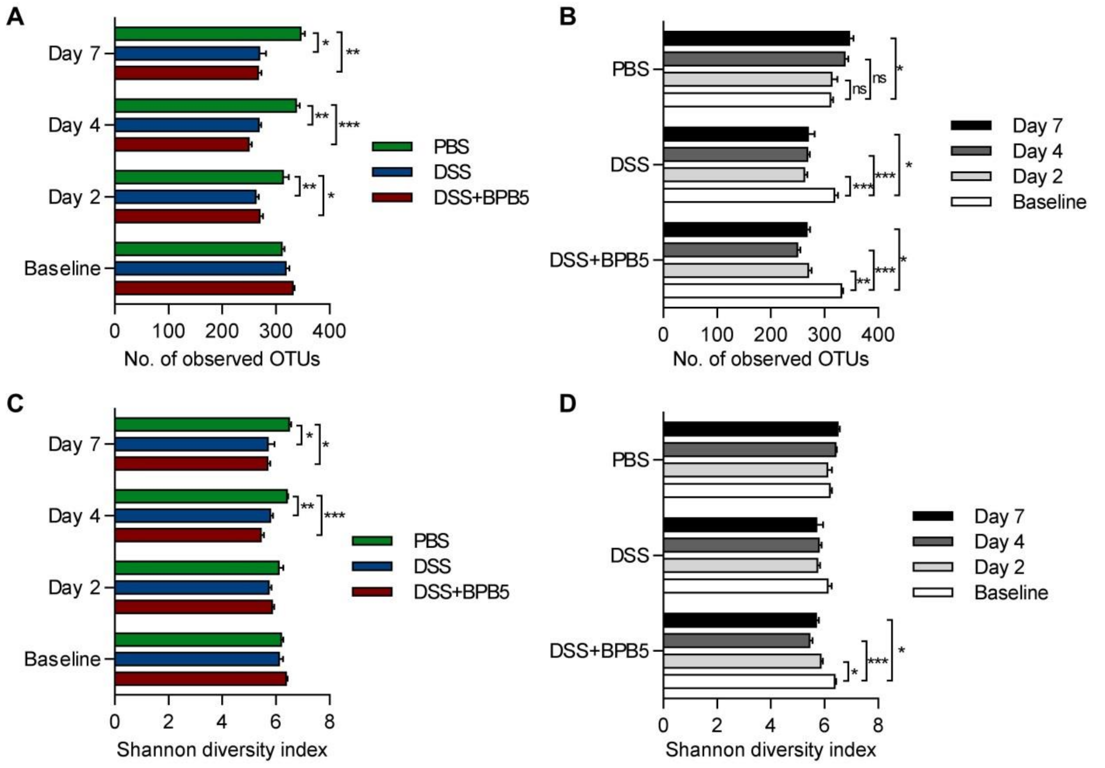

Supplementary Figure S4 OTU-level rarefaction (observed OTUs) (A, B) and Shannon diversity index (C, D) at the sampling level of 9000 of gut microbiota in DSS and/or BPB5 treated mice. The column value was shown as mean ± s.e.m. Kruskal-Wallis test for ANOVA test was used to analyze variation among each subgroup. * $P < 0.05$, ** $P < 0.01$, *** $P < 0.005$. Sample size of each group was as described in Fig. 4C.

Supplementary Figure S5

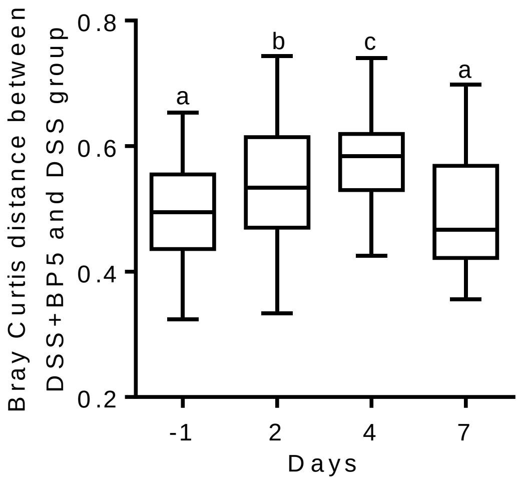

Supplementary Figure S5 Bray Curtis distance between the DSS+BPB5 and DSS groups on Day -1, 2, 4 and 7. Data are plotted as min to max with the median on the line in the box. Values of each group with different letters are significantly different based on the Kruskal-Wallis test (*P* < 0.05).

Supplementary Figure S6

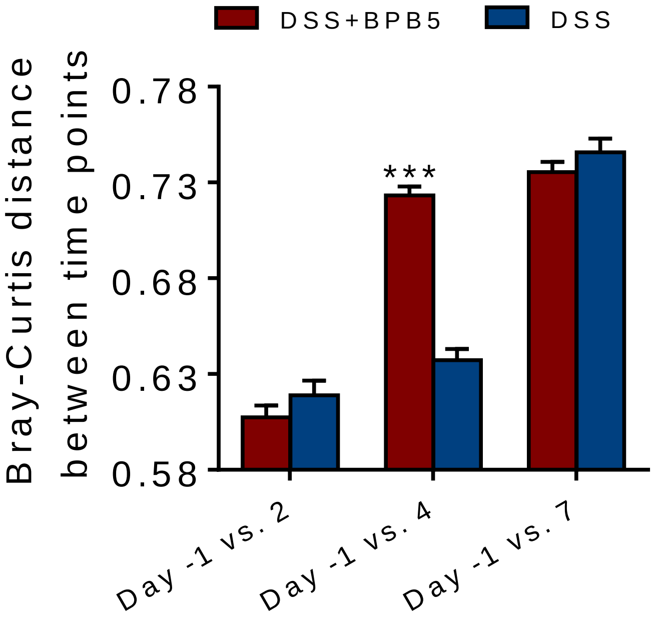

Supplementary Figure S6 Bray Curtis distance between baseline and Day 2, 4 and 7 in DSS+BPB5 and DSS group. Data are plotted as mean ± s.e.m. *** *P*<0.005.

Supplementary Figure S7

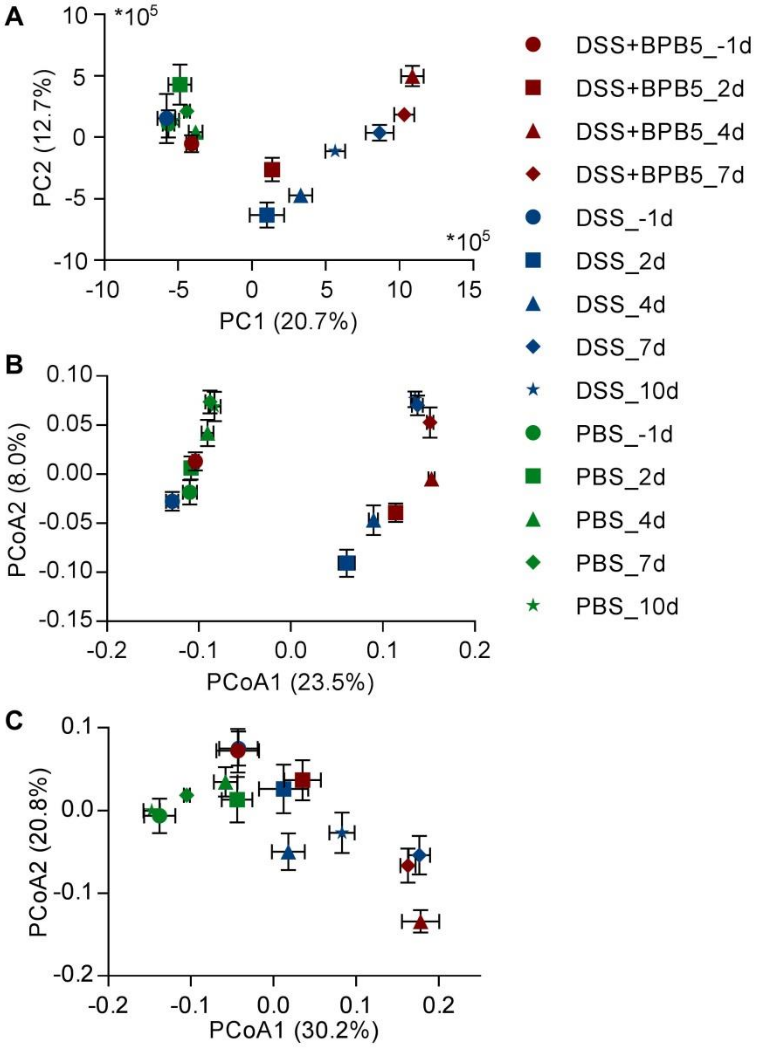

Supplementary Figure S7 Large structural variation of gut microbiota of the 134 samples included in the study. (A) PCA. (B) Unweighted Unifrac PCoA. (C) Weighted Unifrac PCoA. Sample number of each group is the same as Fig. 4C.

Supplementary Figure S8

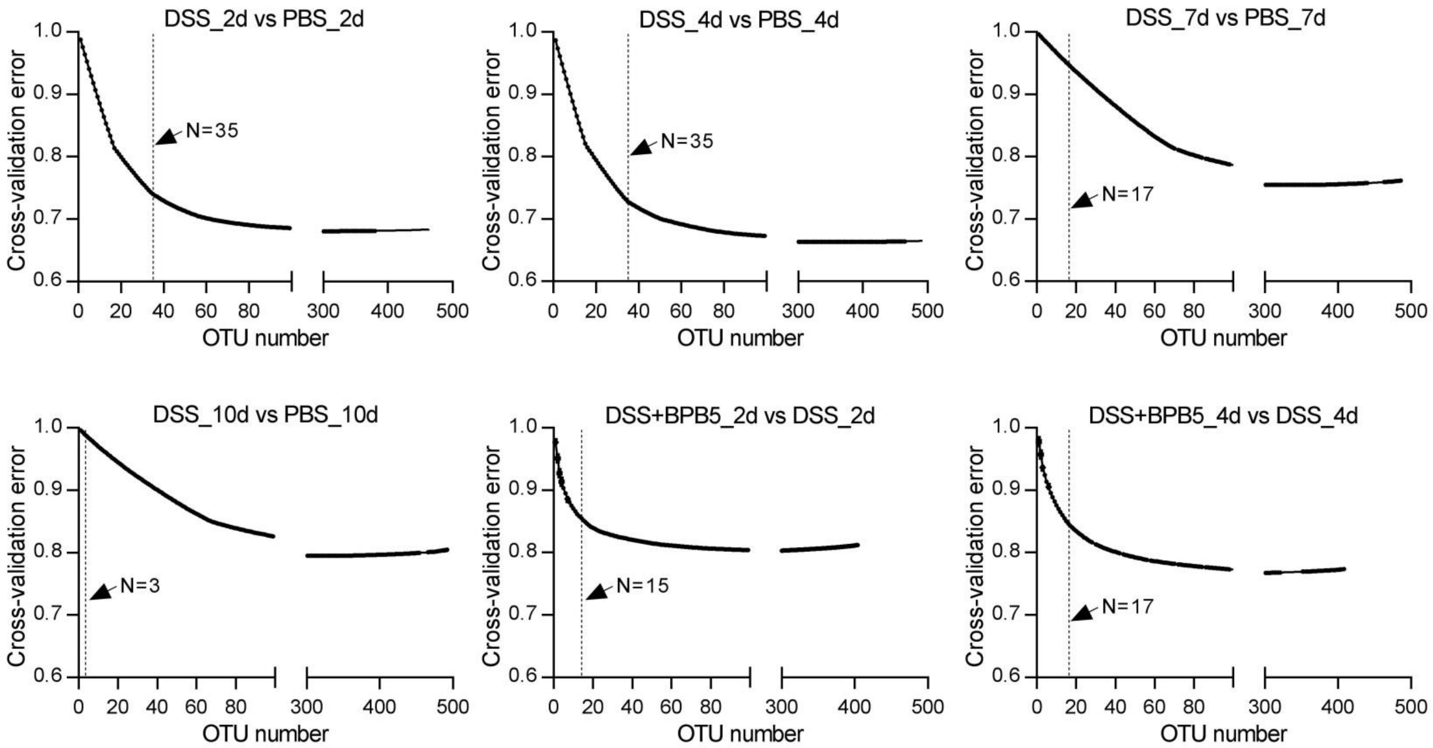

Supplementary Figure S8 83 key OTUs were identified by applying Random Forest classification of their relative abundances in fecal samples, ranked in descending order of their feature accuracy of the models. N is the number of OTUs without which the accuracy of the model will decrease more than 0.3%. The 83 OTUs was the combination of N from 5 models followed by duplicate removal.

Supplementary Figure S9

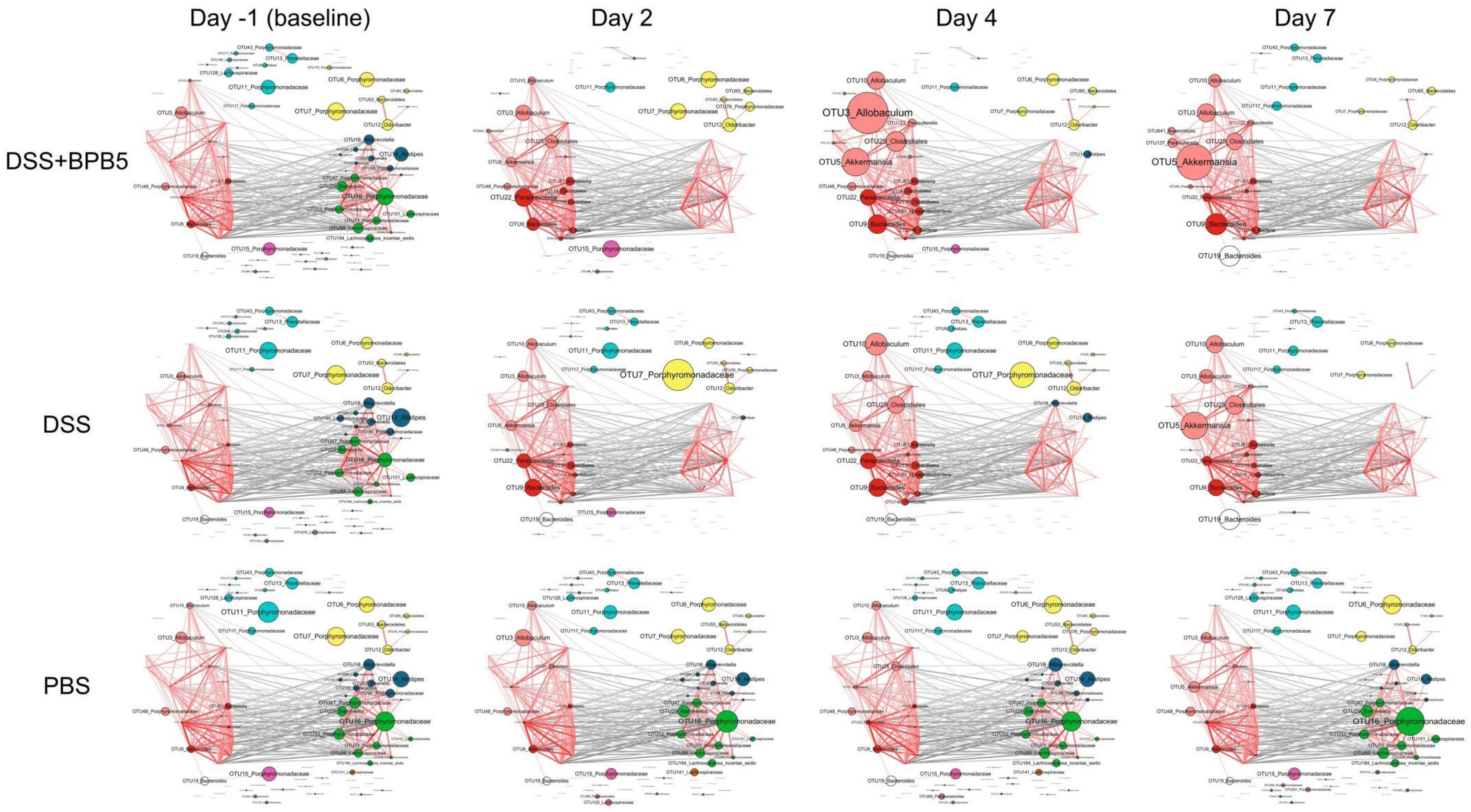

Supplementary Figure S9 Co-abundance pattern of 83 key OTUs which was responsible for the change of gut microbiota induced by DSS and BPB5 intake. The color of the nodes indicates their distribution pattern within the samples. Disc size is correlated with the abundance of OTU. Edges represent SparCC correlation coefficient values above 0.5 (red) or below -0.5 (gray), and width of edges are proportional to the absolute value of R. Sample number of each group is the same as Fig. 4C.
